# Hospital Clowning as Play Stimulus in Healthcare

**DOI:** 10.3390/children1030374

**Published:** 2014-10-30

**Authors:** Laura Anes, Marianne Obi

**Affiliations:** RED NOSES Clowndoctors International, Wattgasse 48, A-1170 Vienna, Austria; E-Mail: laura.anes@rednoses.eu

**Keywords:** illness, RED NOSES clowndoctors, play, humor, recovery, vulnerability, wellbeing, treatment, stimulus, participation

## Abstract

A serious illness, a chronic medical condition or a hospital bed should not deny any child her/his basic right to play, a right essential for children’s development and general wellbeing. In fact, it is in these frightening and anxious moments that play and the stimulus that it provides can help the most. This article will focus on the impacts and benefits of professional hospital clowning for the wellbeing and recovery process of ill and hospitalized children. Our experience has shown that through interactive play and humor, “clowndoctors” can create an enabling and supportive environment that facilitates children’s adaptation to the hospital setting and improves their acceptance of medical procedures and staff. While moving from bedside to bedside, RED NOSES clowndoctors encourage children’s active participation and support their natural instinct to play, fully including them in the interaction, if the children wish to do so. Therefore, clowndoctor performances offer ill children much needed stimulus, self-confidence and courage, elements fundamental to reducing their vulnerability. In this piece, a special emphasis will be put on the various approaches used by RED NOSES clowndoctors to bond and reach out to children suffering from different medical conditions.

## 1. Introduction

The use of humor and performing arts as a complementary therapy in the healing process has become widely accepted, and its benefits are increasingly recognized by health professionals and relevant stakeholders [1]. This growing recognition is directly related to the improvements that have been registered in the quality of care offered to patients in the wards where professional hospital clown programs are being implemented. In a feedback session with the Director of the Psychiatric Department, Beit Jala Governmental Hospital, Dr. Salam Al Kamah stated, “We are very grateful that RED NOSES is in Palestine. Their work is tremendously important, especially in the treatment of children with cancer. They accompany them in their suffering; they are a great support and give them love and affection.”

While RED NOSES programs have gradually developed to encompass new and diverse target groups, such as the elderly and rehabilitation patients, the main focus of this article will be on chronically and acutely ill children. This piece will explore some of the various approaches used by RED NOSES clowndoctors to reach out to and connect with hospitalized and ill children. It will further shed light on the way these approaches impact and contribute to the recovery process and psychosocial wellbeing of the “little patients”.

To meet the needs and characteristics of every specific “audience”, clowndoctor performances make use of culturally-appropriated and specially-designed holistic methods that are essential to connect with the children. During the visits, the perception of the children is profusely stimulated, and their five senses become the central themes. Their natural instinct to play is brought back to life. This impacts the child’s emotional and cognitive development as the clowndoctors provide the space for children to identify and express their emotions, as well as to test and try out their physical capacities in light of certain limitations imposed by their ailment. This is, for example, what is at stake in the Circus Patientus program, where children are provided a stage to express themselves with the assistance of the clowns, as described in Section 2.3.2.

As play is very much about stimulus, during the interaction with the clown, the little patients are encouraged to learn and develop new skills. From the experience of our clowns, detailed in clown reports and regular feedback, as well as supervision sessions, many of the hospitalized children with whom they have contact are, for the very first time, actually exposed to the world of performing arts. In many cases, children take interest in this artistic field and are inspired to learn the tricks performed by the clowns. In the period until the next clown visit, they practice the antics, imagine stories and scenarios that prepare them for the next clown visit. This gives the children in the hospital something to look forward to and allows them to exercise their play predispositions, thus distracting them from the situation of hospitalization that they are in. As described by the Director of Wroclaw Hospital in Poland, Dr. Janusz Jerzak, “Children look forward to every meeting, and for a moment, they forget their illness. The clown visits bring a great deal of positive energy, a great deal of joy and impressions, irrespective of the ages of the children.”

This stimulus offered by the performances gives children in the hospital much needed self-confidence and courage, elements fundamental to reducing their vulnerability. The stimulus and playfulness brought into the hospital room further contributes to easing the fear and stress caused by illness, long hospital stays and/or painful treatments. This is possible because during the interaction with their patients, RED NOSES clowndoctors create an enabling and supportive environment that encourages and promotes the children’s active participation.

## 2. Results and Discussion

Children are one of the most vulnerable groups and are especially sensitive in the hospital environment. Thus, in this distressing period, special emphasis needs to be placed on the emotional and psychosocial support given to the little patients and their relatives and on the need to create enabling environments that allow for children to be children, even in the face of serious or life-threatening illness.

Even if hospital clowning cannot alter the reality that the children have to face, it can lighten their experience and facilitate their adaptation to the hospital environment. Equipped with an abundance of humor, RED NOSES clowndoctors take on the challenging task of conquering fear and emotional stress, which can be detrimental to the healing process. By turning the performance into a restorative experience and not merely a recreational moment, clowndoctor performances seek to attenuate the tension and vulnerability to which children are exposed, giving them vital psychosocial support by applying a great stress reliever and a powerful pain medicine—laughter!

Therefore, we will start by presenting the main concept behind RED NOSES regular children’s programs. This was the first format to be implemented within the group and remains a main pillar of our work. The establishment of RED NOSES Clowndoctors by Monica Culen, Executive Director, and Giora Seeliger, Artistic Director, in 1994, brought about the first children’s programs to be implemented in Austria. They were developed to address the individual needs of children in the hospital with a two-fold aim: to improve the wellbeing and medical condition of ill children and to assure that all children, regardless of their ailment and/or background, are guaranteed their right to play and to take part in cultural life, as enshrined by Article 31 of the United Nations Convention on the Rights of the Child [2].

The success and positive resonance of this regular program resulted in several spill-overs that shaped new formats addressing hospitalized children’s specific needs. That is how programs, such as Circus Patientus, NOS! (Na Operační Sál! (“To the Operation Theatre”)) and others that are the focus in this article, were born. The start of many of these new programs was possible due to the solid collaboration between our clowndoctors and the medical staff in all 187 children’s hospitals in the 11 countries where we currently work. After hospital visits and feedback sessions, they together identify the needs and opportunities for the use of hospital clowning, which benefit ill children. Ongoing comments from medical staff in a major hospital in New Zealand reported that clown visits are not only great for staff morale, but they also made a huge difference to the treatment of the patients.

Based on the different format designs, the article will outline how the various approaches influence children’s recovery processes and how they open opportunities to play and stimulus in an environment that often does not favor the children’s basic instinct to play. Our clowndoctors’ task is to create an enabling, participatory and creative environment that reduces children’s stress levels and challenges them to find tools to cope with their medical condition through interactive play.

The authors of this article stress that the results shown are based on both empirical experience and the ongoing feedback from medical staff, patients and their relatives. They are not the result of any form of scientific research.

### 2.1. Training and Methodology

Working in a very sensitive environment, RED NOSES develops and maintains high ethical and artistic quality standards in all its clown programs. The artist’s work is preceded by rigorous training within the organization, mainly in its training platform—the International School of Humor—located in Vienna, but also through local workshops and exchange programs between partner organizations.

To become a RED NOSES clowndoctor, the candidate has to fulfil the ensuing entry requirements: a minimum age of 23 years; be a professional artist with a background preferably in the performing arts; and a clean police record. After 3–4 auditions, they enter a six-month trial training period, after which, the clown moves into the next training stage for one year.

The ongoing training in three main fields, namely artistic, medical and psychosocial studies for clowns, is given both at the beginner and advanced level. Roughly 16 workshops held per school year focus on different aspects of hospital clowning and are taught by internationally-renowned teachers. Some workshops include practical training in the hospital setting.

After talks and mutual agreements have been reached with the hospital management, the hospital writes an invitation, and a Memorandum of Understanding is signed. Thereafter, active cooperation is started with the hospital. Regular internal feedback sessions allow the hospital and the clowns to exchange information and evaluate the progress of the visits, plus the impact on the hospitalized children.

### 2.2. Regular Visits to Pediatric Wards

As a best practice, regular clowndoctor visits (“clown rounds”) to pediatric hospital wards last between 3 to 5 h and are carried out by two artists, preferably, and when possible, always a male and a female. Depending on the audience, the clowndoctors make use of an array of artistic techniques that range from music and story-telling to circus art, pantomime, magic, improvisation, etc. The type of humor used is also adjusted to the target audience(s) and to their respective situation. Frequently, the clown mirrors the mood and type of humor expressed by the child as a way to start the interaction.

During their visits, the clowns move from bedside to bedside in several children’s wards, from oncology to cardiology, transplant units, intensive care, neonatology, and others. When the clowndoctors enter the room, the child is often alone or with her/his parents. Without playmates or toys, the child is very much restricted in the normal forms of play and in the amount of play possibilities. Therefore, the clowndoctor assumes the role of the playmate and starts a game that opens up and creates an atmosphere of playfulness.

In an environment where the theme of play is lacking, clowns often use the situation of simply having the patient in a hospital room or having medical material and instruments at hand to initiate and engage in some form of play. They create humorous and amusing scenes, where medicines are magic pills, where a hospital bed is a space machine, a flying car or a magic carpet. By turning the hospital room into a space that favors play and by stimulating children’s senses and capabilities, clowndoctors turn the hospital environment into a less scary and more humane place for them. All of the play is adapted to the condition of the child; hence, if the patient is limited to a bed and her/his movement is restricted, physical play is put aside and gives place to verbal play or simply “make believe” situations produced by the clowns. The goal is always to reduce the patient’s anxiety level and to generate a better, more relaxed and calm atmosphere that can boost a child’s recovery process.

The interaction between the clowndoctors and the children creates a supportive environment, during which children are fully included, giving them self-confidence and courage, an element fundamental to their wellbeing. In fact, encouraging and facilitating children’s active participation in the programs and being sensitive to their condition is central to reducing their vulnerability and increasing their reaction. This interaction can also have a non-formal educational component, as the clowndoctor helps children to develop certain skills and understand the need for certain medical procedures, such as the need for taking medicines. Simultaneously, it provides a sense of “normality” through self-expressing activities that allow children to do what they do best—play!

Children’s response to clowndoctors is generally very positive, as portrayed in several written letters from parents we have received and in written reports from our clowns. This response is so positive, that medical staff and family members request the clowns to be present in numerous medical procedures and other delicate moments, like in our special program, NOS!, described later in this article. Naturally, the predisposition to engage in some sort of playful activities varies greatly with the age group, it being easier to engage smaller children than teenagers. However, our experience has shown that even when it takes longer to engage the child, at some point, they respond to the clowns and start taking part in the interaction. From the moment they let the clowns in, a change in their mood is immediately noticeable, and children’s attitudes towards certain medical procedures and staff can improve for the better. A nurse from St. Joseph Krankenhaus, “Josephinchen,” (Children’s General Hospital) in Berlin-Tempelhof said in a feedback session, “Their intervention with the patients was astonishing. Some even forgot that they were in the treatment room. And even for us, the medical staff, it’s a good therapy. We work in a much better mood, and our work with the patients is easier”.

A small-scale research project conducted in cooperation with our partner organization in Slovakia, at the Oncology Clinic for Children in Bratislava [3], showed that ČERVENÝ NOS Clowndoctor programs contributed to the improvement of the emotional mood of hospitalized children. The collection of data was done via individual questionnaires comprised of 10 questions to assess the following variables: mood, adaptability to hospital, importance of the clowndoctor program and impact of the performances. The questionnaire was presented to 38 respondents (16 children aged between 1.5 and 8 years; 16 children from 9 to 16 years of age; plus six mothers). In the case of pre-school age children, the parents responded to the questionnaires after consulting with the child. Older children answered the survey themselves. The parents responded to the questions specifically addressed to them. The answers were recorded by a psychologist.

Differences in the answers of younger and older children were expected, as their needs and reactions to hospitalization vary greatly. The answers of younger children show a higher frequency of positive evaluation in all questions than the responses by older children. For example, when questioned about the influence of clowndoctor visits, 93% of children aged between 1.5 and 8 years say that clowndoctor visits improve their mood and overall state of wellbeing, while that number decreases to 64% among children aged 9 to 16. This can be related in part with older children’s attitudes that perceive this kind of humor as being for young children and not for them anymore. The percentage of negative answers to the 10 questions presented was insignificant. From the results, patients and parents generally agree that the visits by the clowndoctors improve the mood in the room (91%–100% of the respondents) and can offer some practical solutions and tools to face problems common to hospitalized children.

In fact, through light-hearted play, professionally trained hospital clowns provide children in hospitals with coping strategies. According to RED NOSES International expert, Gary Edwards, there are five distinctions in child developmental stages that are relevant for clown work in hospital—infants, toddlers, pre-school children, school-aged children and young persons. Each stage requires specific artistic approaches to achieve the expected result of the visit (as described above). For example, to ease a toddler’s experience in the hospital and reduce fear, the clowns often resort to some sort of play to distract the child. In this case, bubbles and post-procedure play can work very well. Role playing can be a powerful tool to divert the attention of toddlers, pre-school and school-aged children from certain medical procedures. It provides an alternative focus during the procedure, which will not be associated with a painful experience, but rather with a positive experience of interaction with the clown. Young persons are more hesitant to express their feelings of pain and do not want to be embarrassed. They also take more personally the stigma of being different and isolated from their friends. In this case, the clown is very attentive to respect young persons’ privacy and always avoids being condescending during the interaction. It is equally very important to include these young adults in their treatment.

The clowns are also very successful in involving the child in the treatment process, which she/he does not usually understand and/or accept. Clowndoctors are very successful in conveying content that will be essential for treatment, especially for children with chronic diseases, such as the need of taking medicine or undergoing regular therapy. Therefore, children have something to look forward to, and while waiting for the next regular visit, they attempt tricks and are immersed in the world of play once again.

Feedback from hospitals and medical staff regarding the contribution and benefits of regular visits by professional clowndoctors to the wellbeing of children in the hospital has been equally tremendously positive. To maintain the high artistic and quality standards of the programs, these feedback sessions take place on a regular basis as per the Memorandum of Understanding with the hospital. They are put in writing by the clowns, reported back to the management of RED NOSES and are essential to assess the impact that clown visits have on the patients and to look for common areas for cooperation. This has resulted in an exponential increase of the number of children’s wards and patients visited by RED NOSES clowndoctors. In 2013, we visited over 485,000 children in 187 hospitals in 11 countries. The success of the program is also translated in the number of requests to open a new program or to increase the number of visits to pediatric wards.

#### Regular Visits to Hospitals in the West Bank

Regular visits to pediatric wards, which have successfully been implemented throughout Europe, have also proven to be very successful in other areas of the world. A good example of best practices is the RED NOSES regular children’s program in Palestine, implemented by our branch office in the West Bank [4].

**Figure 1:**
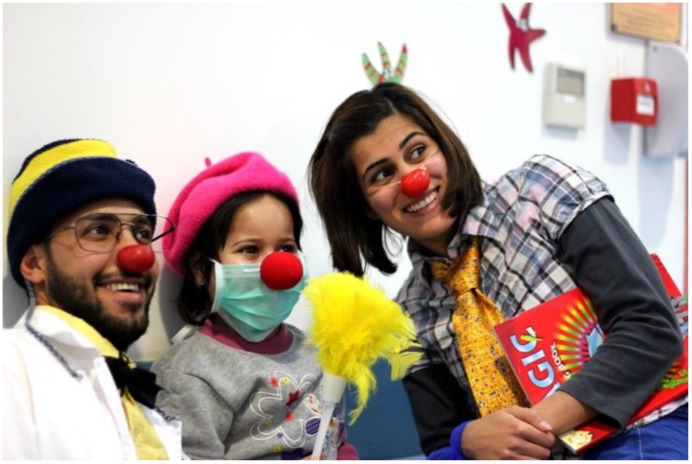
Regular children’s program in Beit Jala Governmental Hospital.

Recognizing the needs of the population in the West Bank and the Gaza Strip, RED NOSES International and RED NOSES Palestine have put in place a regular program for children in hospitals, as the one described above in Section 2.2. This program follows the same approach as the one implemented by our partner organizations in Europe and New Zealand. Currently, these visits are being implemented in the four partner hospitals in the West Bank—Beit Jala Governmental Hospital (Figure 1), Ramallah Medical Complex, Hebron Governmental Hospital and Augusta Viktoria in Jerusalem—with incredibly positive feedback from patients, relatives and medical staff. The success of our program in the Palestinian territories demonstrates that hospital clown programs can be implemented everywhere in the world with the same positive results as the ones already proven in the West.

RED NOSES programs in the West Bank hospitals not only bring Palestinian children in the hospital a much needed smile and play opportunities, but they also help undermine the stress caused by the extremely aggravating endeavor of accessing health services. Regular visits to these four hospitals also present an element of stability in an area where family visits cannot be taken for granted. In a territory deeply affected by the restriction on the freedom of movement, due to the permit system in place in the Palestinian territories, “mothers below the age of 39 and fathers are the most frequently denied permits” [5]. This absence can intensify the feeling of insecurity and can induce further stress, discomfort and malaise, especially among children.

In addition, Palestinian children continue to register high levels of post-traumatic stress (PTS) and various mood and anxiety disorders, which render essential programs focusing on easing the stress caused by illness and the violence in their surroundings. According to UN agencies, 14.2% of kindergarten-aged children present indications of emotional stress [6] related to family violence (which tends to increase in cases where unemployment and poverty are present) and with the conflict (many children suffered or witnessed violence and/or injury, several types of ill-treatment and abuse, arrests, demolition of their houses, settler-related violence, *etc*.) [7]. By focusing on psychological wellbeing, RED NOSES programs help these children to cope with the stress caused by the violence in their surroundings, as clowndoctor performances provide a platform for children to handle daunting situations. Clowndoctors reach beyond the moment of interaction; they help build human capacity to deal with illness and disability. Through humor, clown work promotes critical thinking and encourages children’s ability to understand their situation and to look for ways to challenge it.

Moreover, the implementation of this program helps the Palestinian health system to respond to several immediate challenges, including an epidemiological transition that is translated in an ever-increasing incidence of chronic and non-communicable diseases (NCDs) among Palestinian children [8]. With several patients having to cope with long stays at the hospital and eventual long processes of medical referrals for treatment outside of their area of residence or abroad, the psychological burden of these diseases cannot be overlooked.

RED NOSES visited over 11,500 Palestinian children in the West Bank in 2013. Half of the Palestinian population is under 16 years old, and according to the Palestinian Central Bureau of Statistics (PCBS), the percentage of children aged between 0–14 years is 40.1% of the whole population (38% in the West Bank and 43% in Gaza) [9].

### 2.3. RED NOSES Special Programs

#### 2.3.1. Caravan Orchestra: Empowering Children with Disabilities

In line with Article 30 § 5.d. of the UN Convention on the Rights of Persons with Disabilities [10], RED NOSES recognizes the necessity for children with disabilities to participate in play, recreation and leisure.

2014 marks the second anniversary of the RED NOSES program, the Caravan Orchestra, based on the format from the CliniClowns Foundation in the Netherlands. This tailor-made musical theatre program is uniquely designed for children and youth with mental and multiple disabilities.

The performance: A group of three specially trained clowns travel from “oasis to oasis” visiting institutions for special education. They are looking for new musicians to join their orchestra, because music is their passion. The clowns quickly find what they are looking for: the children in the room. A treasure chest reveals keys that become musical instruments; the children’s names are woven into highly personalized songs, and everything together results in a joint melody.

At the heart of everything, the child: For one week, they immerse themselves into the children’s world and reach out to them, individually relating to their respective needs. During the workshop, the clowns concentrate on the receptiveness and emotional status of the participants, approaching them in a variety of holistic ways, with music, colorful requisites, innumerable sounds and, of course, the playful art of clowning. Every child reacts differently. Some participate immediately, clap and laugh, while the reactions of others are subtle. All of the senses of the children are stimulated, and their creativity emerges during this personal psychosocial intervention.

Through interactive play based on sensitivity and respect, the clowns use humor and laughter as valuable tools to encourage and empower this vulnerable group, whose self-esteem and self-confidence become enhanced, and a good state of wellbeing emerges. The fight against social stigmas and discrimination is promoted, enabling the individuals to participate and integrate better within the hospital environment and the community.

Having received good resonance in Austria, we now have the program running in four more of our eleven partner organizations in the Czech Republic, Hungary, Slovakia and Slovenia. In 2013, over 120 plays that included 880 children and youth with disabilities were performed.

A special team of five experienced clowns (three main + two in reserve) are given extra artistic training in musical instruments, sign language and improvisation in order to deal with the different disabilities of the target group. The training and the first three performances are overseen by the project leader [11].

#### 2.3.2. Circus Patientus: The Circus Has Been a Guest in Hospitals Since 2004

The Circus Patientus program has its roots in the Czech Republic’s ZDRAVOTNÍ KLAUN, RED NOSES International partner organization, which counts on the support of the Czech Chapter of the United Nations organization, the World Health Organization (WHO). The first hospital circus performance took place in the Czech Republic in 2004 and catered to young, long-term care patients in oncological and orthopedic units and pediatric and adolescent psychiatric facilities.

All sick children who want to participate are welcome. The circus program is customized and rehearsed with children no matter what their physical state—in a cast, bedridden, with crutches—everything goes! Together with two RED NOSES clowns, the children convert the hospital room into a stage, where the children are transformed into artful magicians, dazzling artists, skilled unicycle riders and muscular weightlifters. At the end of the project week, the grand performance in front of doctors, nursing staff and family members is met with thunderous ovations.

The sick child is relieved of his or her passive role in the everyday hospital routine, even if their illness, concerns and disorders only temporarily fade into the background. As full attention is focused on their new skills, the young patients discover a new side to themselves. The round of applause boosts their self-confidence and the creative collaboration stimulates their enthusiasm and vitality.

**Figure 2:**
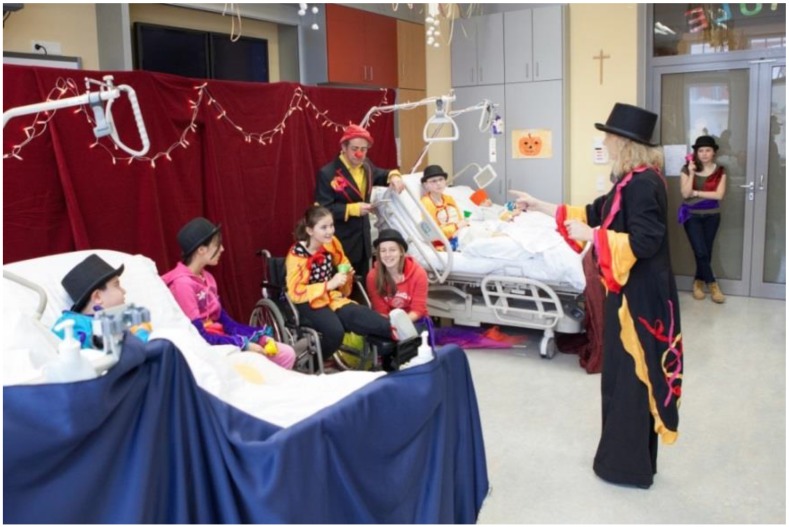
Performance of Circus Patientus in Austria.

Young long-term care patients in the Czech Republic, Austria (Figure 2), Slovakia, Slovenia and Lithuania are transformed into enthusiastic circus ring stars. In 2013, we brought the magic of performances to more than 2,200 children. In several cases, they were given the opportunity to create their own play script, exploring their abilities to the maximum and making them the stars of the show!

**Figure 3:**
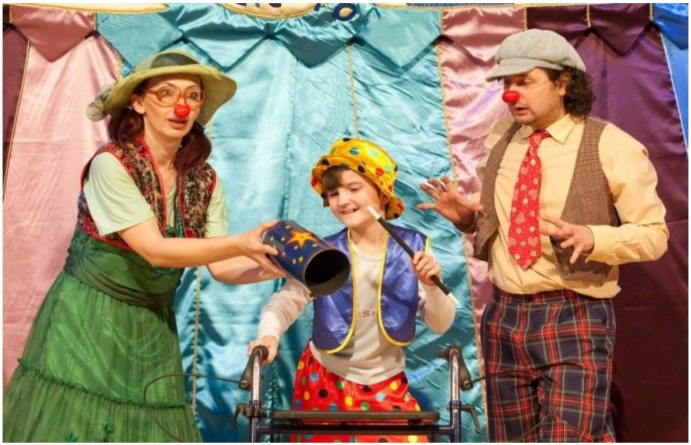
Performance of Circus Patientus in Slovakia.

In the Czech Republic and Slovakia (Figure 3), our RED NOSES team even puts together a tent that literally brings “the circus into the hospital”. Playfulness is triggered, and the magic of the circus tent transports the patients, relatives and medical staff to a world far away from the hospital reality, at least for some hours. Feedback from relatives and medical staff has been tremendously positive.

#### 2.3.3. NOS! (Na Operační Sál! (“To the Operation Theatre”))

**Figure 4:**
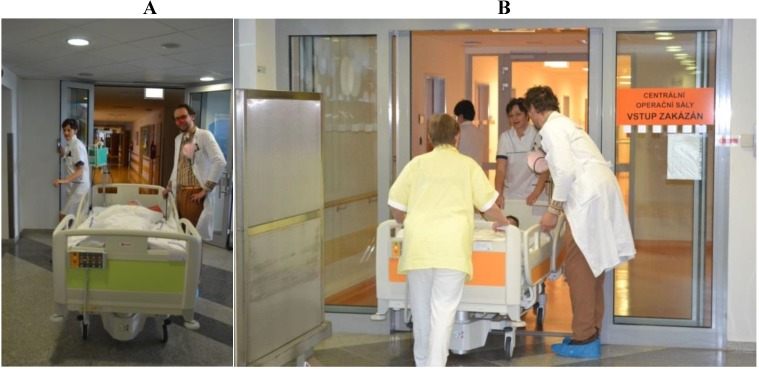
Lukáš Houdek, a.k.a. RED NOSES, clown Dr. Ivan Motyka (Zdravotní Klaun, CZ) helps patients preparing for surgery.

In 2013, another very successful Czech pilot program for humor intervention in surgery preparation—NOS!—started at the children’s surgery department in University Hospital—Motol, Prague and shortly afterwards in the Ostrava-Poruba Hospital; and since 2014, in the Teaching Hospital Hradec Kralove (Figure 4A,B).

In this intervention, the clowns are the trigger for humor, which takes place in this environment that is normally not conducive to play in.

One experienced clowndoctor spends time with a child and the parents during preparations for an operation, assuaging their fears, anxiety and insecurities during these difficult moments through sensitive, humorous intervention. Under these specific conditions, a single clown can better exercise his or her artistic empathy. For the intervention, the clown identifies several stress moments—changing scrubs, coming into the department, entering the “staff only area”, waking up in a new room—and replaces them with playfulness to help ease the stress of the patient.

The actual time the clown spends at the ward varies according to the number of children for surgery, so the visit can last between 4 to 6 h. When children come back from surgery, most of them are still asleep, and the nurses insist that they sleep as long as possible, advising parents not to interrupt their sleep. When they do wake up, quite often, they have an adverse reaction to the anesthetics (crying, fits of anger). During these episodes, not even a mother’s attention helps. The clown’s role is usually to make it obvious to the parent that he or she is available to help with the child, but leaves it up to them to choose the appropriate moment. The child falls asleep and wakes up with the same clown and play taking place. A seamless overlap has occurred without the child ever noticing any interruption.

After an internal evaluation period of three months, a meeting was held in the hospitals with the relevant hospital staff. In all three hospitals, the reception of the project was very positive. This project requires close ties, cooperation and trust among the participating clowns and all staff members. In fact, the effect of the clown’s presence at the ward seems to have achieved a subjective feeling of more “room,” more calm, more relaxation and more fun. We also received several spontaneous and hugely positive feedback letters from parents, such as the mother of a small patient (name withheld) who said, “My son was crying and didn’t want to swallow the oral anesthetic. He made such problems, I was so nervous. I was so happy that the clown was here and able to sooth his nerves, as well as mine! He really saved the situation!”

The project has, in the meantime, extended to medical facilities in Slovakia, where the clowns now accompany children into the surgery room for long, painful medical procedures. Last year, we performed 34 of these visits, during which RED NOSES clowns attended to 333 children.

#### 2.3.4. Clown on Duty

In 2010, RDEČI NOSKI in Slovenia began a project that has been in operation ever since: Clown on Duty. This project was started at the request of several nurses and is still very successful. Over a span of a few days, the clowns participate in the entire hospital routine, from A to Z: during medical treatments, such as drawing blood or taking X-rays, during boring waiting times, during preparatory procedures before an operation, *etc*. During this entire period, the clowndoctors are present around the clock for patients, just like physicians and the nursing staff. Their presence brings a sense of security and variety.

The project, which takes place once or twice a year, builds bridges between the hospital staff and the clowns. During the project, physicians and nursing staff can learn from the humorous interaction with patients, and the clowns gain new and important experience that helps them in the everyday hospital setting. The goal of this partnership is to facilitate exchange between artists and medical staff, but also to create a more humane and patient-friendly atmosphere in hospital wards. The immediate result of the clowns’ presence is a lighter and more cheerful mood, a change that is beneficial for both patients and medical staff who deal with the burden of disease, pain and loss on a daily basis.

#### 2.3.5. “Wake Up. Come Back to Us!”: Visits at the Bedside of Pediatric Coma Patients

Faced with craniocerebral trauma or other such life-threatening diagnosis, children in hospital need very specific neurological treatment and special care during rehabilitation.

To serve exactly this purpose, the “Budzik Klinik” (“Alarm Clock Clinic”) in Warsaw opened its doors in summer, 2013. The first and most modern of its kind in Poland, the clinic specializes in free treatment and follow-up for young coma patients.

The hospital “Budzik Klinik” has 15 beds on three floors and provides an advanced medical infrastructure for affected children. An important part of the therapy is the fact that parents are always allowed to be present. The founder, Ewa Blaszczyk, actress and concerned mother, built the clinic with the help of her charitable foundation, FUNDACJA Ewy Błaszczyk “AKOGO?” and many generous donors and sponsors.

In addition to doctors, neurologists, physiotherapists and psychologists, the Polish RED NOSES organization, CZERWONE NOSKI, was also asked to be part of the treatment plan from the outset, because their presence is an important stimulus for comatose children. Clown work performed directly at the bedside is an important and powerful stimulus, not only for comatose children, but also for those who have come out of a coma and gone into rehabilitation. Therapists in the clinic involve the clowns in all of the stages of the recovery process.

RED NOSES has been visiting little patients in the “Budzik Klinik” on a regular basis since August, 2013, and a unique and highly successful project has developed.

Even if it seems that the children do not notice or respond to anything, the clowns contribute greatly to the waking up process with their sensitive, poetic and musical visits. They are valuable companions, especially when the little ones gradually regain consciousness.

Eight children have already woken up from being in a coma (a great success for the clinic). Additionally, even afterwards, RED NOSES clowns are still warmly welcomed by the little patients and their parents. The humorous interaction supports their rehabilitation and helps all those involved to endure and keep on going.

When the little patients finally leave the hospital, they see the clowns as close friends and are happy to stay in touch with them. They remain by their sides to accompany them on their journey back to life, giving them hope and courage.

#### 2.3.6. “We Have Slippers”

Palliative medicine utilizes a multidisciplinary approach to patient care, relying on input at different levels from doctors, pharmacists, nurses, chaplains, social workers, psychologists and other health professionals. Together, they formulate a plan of care to relieve the suffering in a patient’s life. The philosophy behind palliative care lies in holistically addressing physical, emotional, spiritual and social issues.

According to Gary Edwards, RED NOSES expert, tearing down the stigma in palliative care is one of the benefits of the RED NOSES children’s program, “We have slippers,” which he started in the Czech Republic in 2011. Children often do not understand the processes taking place around them when in palliative care. They do not understand their illness and the limitations and restrictions involved in their situation. They do not understand the reason why they are isolated and why the entire mood in the room is somber and sad; why everyone is quiet; why they cannot play. They often feel that it is their fault, that they are being punished, since they are not allowed to play. This common notion goes against the natural instinct of the child, who has the right to play in any situation. Children are very sensitive and can feel the atmosphere is daunting and stressful for their family members.

The clowns who carry out this program, which was developed together with a mobile hospice team, are specially trained in visiting the patient at home and dealing with the implications that are involved in such circumstances. The biggest problem to implementing this program is that there is a need for the parents to initiate and ask for it, since it is a very personalized form of intervention. The parents have to realize that the stimulus for playfulness is lacking, needs to be present and needs to be filled. The visit is therefore planned in close cooperation with the parents, as it has to be a more compassionate visit responding to human needs.

The clowns come into the house and initiate play, quite often in the context of a birthday party; or sometimes, the family just invites the children’s friends over, and this works very well, giving it the feel of a real party.

However, the intervention is often a one-time visit when the clowns join a house call by the doctors. They drive in the same car to make sure that when the doctors are there, the clowns are also present. The duration of the visit always varies, as there needs to be space for some fun. This requires good cooperation with the doctors, who understand that they need to exercise a lot of patience, since it is not a quick house call.

Due to the success of the program for children, medical staff asked for it to also take place in palliative care for Czech adults.

#### 2.3.7. Emergency Smile

In recognizing that opportunities for play, recreation and cultural activities can have a significant therapeutic and rehabilitative role in helping children to recover a sense of normality and joy after their experience of loss, dislocation and trauma, RED NOSES International created the “Emergency Smile” project in 2012. This program builds upon the previous international engagements of RED NOSES, including the disaster relief project in Kosovo’s largest refugee camp in 1999 and the emergency response following the 2011 Christchurch earthquake in New Zealand.

**Figure 5:**
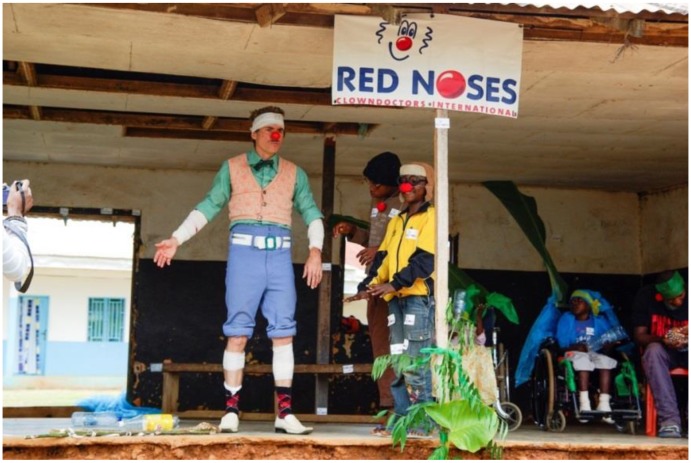
Final performance of “Emergency Smile” in Akonolinga, Cameroon.

The new program had its official debut in the summer of 2013 at a medical facility for tropical diseases in Akonolinga, Cameroon. In a pilot project initiated in partnership with Médecins Sans Frontières (MSF), RED NOSES sent a small team of clowns to raise awareness locally and contribute to managing the pain plan of patients with the stigmatized and neglected disease, Buruli ulcer. During their three-week long stay, the clown team visited the ward twice a day and took their juggling acts and funny songs to the dressing rooms, physiotherapy sessions and into the waiting room of the operating theatre. At the end of the trip, the clowns helped the children on the ward put on a play about early detection of the disease, conveying a clear message that the best treatment is found in the hospital (Figure 5).

Through educational theatre, our clowndoctors actively worked to demystify this neglected tropical disease and to break away from the social stigmas that are associated with Buruli ulcer. For example, the clowns actively contributed to integrating a boy found with advanced Buruli ulcer in a rural area and who was abandoned by his family because of the disease. During the visit, the boy was amazed by the clowns, whom he followed everywhere. Because of his dedication, our team chose him to be their helper and assistant in the hospital throughout the RED NOSES stay. After our departure and in contacts with medical staff in Akonolinga, we were told that the boy was now a very confident boy, proud of his newly acquired status as “assistant” of the RED NOSES clowns. He was no longer the boy that was abandoned because of his disease, but rather the “clown helper”. The presence of the clowns was a trigger that boosted his confidence and has, since then, helped to lift his spirits and to face the painful disease.

In addition, according to the feedback reports from the local medical staff, the impact of the RED NOSES visits on the patients’ wellbeing was astonishing. Worth noticing is the positive resonance of the presence of the clowns in the treatment room while patients undergo the painful procedures of changing band aids. This process was described by the medical staff as very painful and traumatic, especially for small children. When the clowns were present during this procedure, the children were distracted by the cheerful figure of the clown that helped shift their attention from the procedure itself, with amazing results that impressed the medical staff to the point of having them ask for basic training in hospital clowning, in order to apply some of the successful tricks after the departure of the clowndoctors.

## 3. Conclusions

Psychologically, hospital clowning has proven essential in providing children with coping strategies and in contributing to raise their self-esteem, build up confidence and improve their social abilities. Through interactive play, RED NOSES clowndoctor performances support resilience, reduce patients’ anxiety and seek to have a positive impact on the overall wellbeing of children suffering from ill health. Additionally, this patient-oriented care has a very positive impact on the parents and the way they deal with the health condition of their children.

The success of performances is linked not only to the high quality and specialized artistic training of the performing artists involved, but also to the interdisciplinary and cross-sectorial approach adopted in the implementation of hospital clowning programs—an approach that strengthens the linkages between culture, health and development.

Due to the positive resonance from patients and medical institutions, RED NOSES children’s programs have greatly expanded throughout the years. RED NOSES is currently addressing the needs of a growing number of children, having visited in 2013 alone more than 485,000 children in 187 children’s hospitals, in 11 countries. The groups of children visited by our clowndoctors have become increasingly heterogenic, thus requiring specific approaches to respond to their individual needs. The person-centered approach implemented by our clowndoctors has allowed us to reach out to children with diverse medical conditions, from diverse cultural and socio-economic backgrounds and in very different environments and countries. The result is that regardless of the environment and medical condition of these children, hospital clowning contributes to the improvement of their wellbeing and helps fight stigmas against children with disabilities.
